# The effect of PU.1 knockdown on gene expression and function of mast cells

**DOI:** 10.1038/s41598-018-19378-y

**Published:** 2018-01-31

**Authors:** Yoshihito Oda, Kazumi Kasakura, Izumi Fujigaki, Azusa Kageyama, Ko Okumura, Hideoki Ogawa, Takuya Yashiro, Chiharu Nishiyama

**Affiliations:** 1 Department of Biological Science and Technology, Faculty of Industrial Science and Technology, Tokyo University of Science, 6-3-1 Niijuku, Katsushika-ku, Tokyo 125-8585 Japan; 2 Atopy (Allergy) Research Center, Juntendo University School of Medicine, 2-1-1 Hongo, Bunkyo-ku, Tokyo 113-8421 Japan

## Abstract

PU.1 is a hematopoietic cell-specific transcription factor. In the current study, we investigated the role of PU.1 in the gene expression and the function of mouse mast cells (MCs) *in vitro* and *in vivo*. When PU.1 siRNA was introduced into bone marrow-derived MCs (BMMCs), IgE-mediated activation was reduced, and the Syk and FcεRIβ mRNA levels were significantly decreased. As the regulatory mechanism of the *Syk* gene is largely unknown, we performed promoter analysis and found that PU.1 transactivated the *Syk* promoter through direct binding to a *cis*-element in the 5′-untranslated region. The involvement of PU.1 in the *Syk* promoter was also observed in mouse dendritic cells and human MCs, suggesting that the relationship between PU.1 and Syk is common in mammals and in hematopoietic lineages. When antigen was administrated intravenously after the transfusion of siRNA-transfected BMMCs in the mouse footpad, the footpad thickening was significantly suppressed by PU.1 knockdown. Finally, administration of the immunomodulator pomalidomide suppressed passive systemic anaphylaxis of mice. Taken together, these results indicate that PU.1 knockdown might be an efficacious strategy for the prevention of MC-mediated allergic diseases.

## Introduction

Mast cells (MCs) play an important role in IgE-mediated allergic responses^1^. MCs express FcεRI, the high-affinity receptor for IgE, whose cross-linking by IgE and multivalent antigens causes stimulation of cells, including rapid degranulation, immediate eicosanoid generation, and transcription of cytokine genes. In addition to the cross-linking by antigens and IgE, binding of monomeric IgE to FcεRI, even in the absence of antigen, accelerates several biological activities of MCs^2–5^. FcεRI is composed of three subunits-α, β, and γ-and is expressed on the cell surface as an αβγ_2_ tetramer or αγ_2_ trimer^6^. The expression of α and β is mainly restricted to FcεRI-expressing cells, whereas γ is detected in other hematopoietic lineages because of its role as a common component of FcγRs.

To clarify the mechanism of cell type-specific expression of FcεRI, we conducted a study of the transcriptional regulation of *FCER1A* (encoding FcεRIα) and *MS4A2* (encoding FcεRIβ) and identified several transcriptional regulators^7–16^. Transcription factors PU.1, GATA1, and GATA2, and the cofactor FOG-1 are all candidates for determining cell type specificity. Briefly, cooperation between PU.1 and GATAs in FcεRI-positive cells^7,15^ and the suppressive effect of FOG-1 on GATA1 in FcεRI-negative cells^16^ determine the cell type-specific expression of human *FCER1A* and mouse *Ms4a2* genes, respectively. Based on these findings, we analyzed the effect of knockdown of PU.1, GATA1, or GATA2 on the expression and function of FcεRI in human MCs and found that introduction of PU.1 siRNA most significantly suppressed the expression and function of FcεRI due to the substantial reduction of *FCERIA* transcription^15^. These results prompted us to evaluate the effect of PU.1 siRNA on MC-dependent allergic reactions *in vivo*.

In the present study, we focused on the involvement of PU.1 in the gene expression and function of mouse MCs and evaluated the effect of PU.1 knockdown on MC-mediated allergic responses *in vitro* and *in vivo*.

## Results

### Effects of PU.1 knockdown on the gene expression and function of mouse MCs

**Figure 1:**
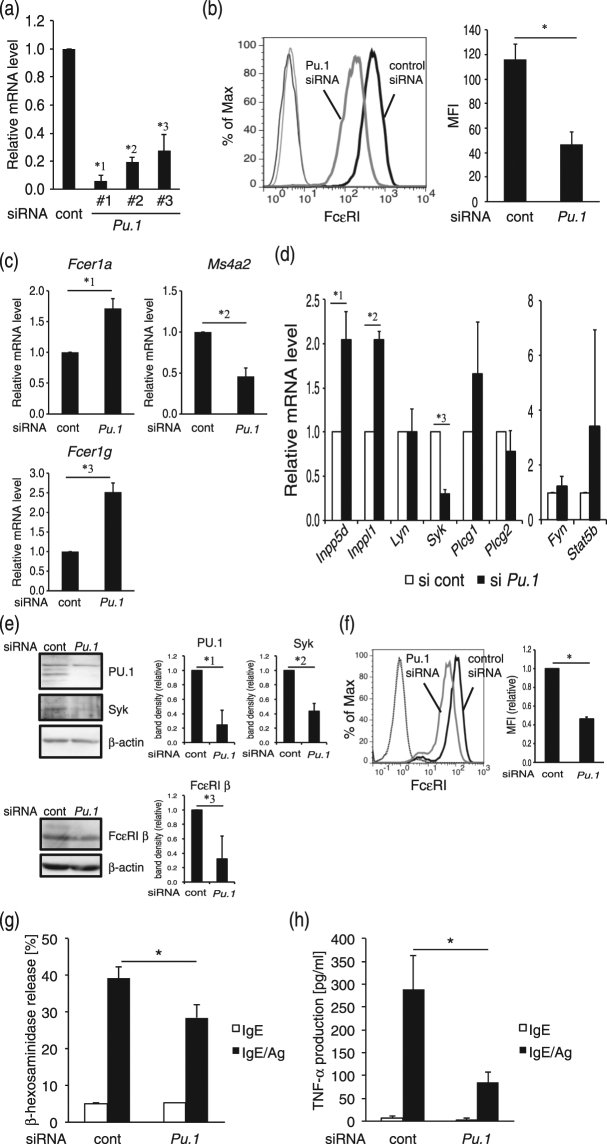
Effect of PU.1 siRNA on mouse MCs. (**a**) mRNA level of PU.1. *1, *p* = 0.00064; *2, *p* = 0.00061; *3, *p* = 0.0011. n = 3–4. (**b**) Cell surface expression level of FcεRI. MFI; Mean fluorescence intensity. *, *p* = 0.000016. n = 5. (**c)** mRNA levels of the α−, β−, and γ-chains of FcεRI. *1, *p* = 0.0000056; *2, *p* = 0.0000017; *3, *p* = 0.00000055. n = 8. (**d**) mRNA levels of intracellular molecules. *1, *p* = 0.029; *2, *p* = 0.0025; *3, *p* = 0.0018. n = 3. (**e**) Western blotting analyses of whole cell lysates. Full-length blots are included in Supplemental Fig. S1. *1, *p* = 0.024; *2, *p* = 0.012; *3, *p* = 0.023. n = 3–4. (**f**) Intracellular staining of FcεRIα. A typical result is shown at left. Quantitative analysis data of MFI obtained in three independent experiments are shown at right. *p = 0.0017. (**g**) IgE-mediated degranulation degree. *p = 0.032. n = 11. (**h**) IgE-mediated TNF-α release. *p = 0.037. n = 3. The data in Fig. 1 represent the mean ± SD of independent experiments (“n” times repeated) performed with duplicate samples.

Previously, we found that PU.1 transactivates the *FCER1A* gene encoding FcεRIα^7^ and that PU.1 knockdown suppresses FcεRI expression and IgE-mediated degranulation of human MCs^15^. In contrast, it was unclear whether PU.1 knockdown affected the transcription of FcεRI components and the subsequent cell surface expression level and function of FcεRI in mouse MCs. Thus, we evaluated the effect of PU.1 siRNA on the cell surface expression level of FcεRI and the mRNA levels of the FcεRI α−, β−, and γ-chains. First, we evaluated the effect of three siRNAs (#1, #2, and #3) encoding different nucleotide sequences of PU.1. As shown in Fig. 1(a), we confirmed that the three siRNAs significantly knocked down PU.1 mRNA, and siRNA #1 was the most effective. Therefore, we used #1 in the following experiments. Flow cytometric analysis revealed that PU.1 knockdown significantly suppressed cell surface expression of FcεRI on BMMCs when the PU.1 mRNA level decreased under 10% compared with that of the control (Fig. 1(b)). Although the suppressive effect of PU.1 siRNA on the cell surface FcεRI level was commonly observed in humans^15^ and mice (Fig. 1(b)), surprisingly, PU.1 knockdown decreased the FcεRI β-chain mRNA level, whereas the mRNA levels of the FcεRI α- and γ-chains increased in PU.1 knockdown cells (Fig. 1(c)). Considering that PU.1 knockdown in human MCs decreased the mRNA level of the human α-chain, but did not affect the mRNA levels of human β- and γ-chains^15^, the role of PU.1 in the transcription of FcεRI subunits appears to be different between humans and mice. To evaluate the effect of PU.1 knockdown on the expression of signal transduction molecules and on IgE-mediated activation in MCs, we determined the mRNA levels of signal transduction molecules, the degree of IgE-mediated degranulation, and IgE-mediated TNF-α release in BMMCs. Using DNA microarray analysis, we found that Syk mRNA showed the greatest decrease in PU.1 knockdown cells (data not shown). Further detailed analysis using quantitative RT-PCR confirmed that the Syk mRNA level was substantially reduced in PU.1 knockdown cells (Fig. 1(d)). We also found that transcripts of the phosphatases SHIP-1 and SHIP-2 were markedly increased by PU.1 siRNA knockdown, whereas the mRNA levels of Lyn, PLCγ1, PLCγ2, Fyn, and Stat5 were not affected by PU.1 knockdown (Fig. 1(d)). Using Western blotting analyses, we confirmed that the protein levels of PU.1, Syk, and FcεRIβ were significantly decreased by PU.1 knockdown (Fig. 1(e)). The staining of permeabilized cells showed that FcεRIα protein levels in PU.1 knockdown cells were lower than those in control cells (Fig. 1(f)), suggesting that the mRNA increase in FcεRIα by PU.1 knockdown was not reflected in the total amount of FcεRIα protein (Fig. 1(c)). The reduction of FcεRIβ protein level may result in suppression of cell surface expression of FcεRIα (Fig. 1(b)) by affecting the formation and/or stability of the FcεRI complex. After down-regulation of Syk expression and cell surface FcεRI and up-regulation of SHIP-1 and SHIP-2, PU.1 knockdown suppressed IgE-mediated degranulation (Fig. 1(g)) and TNF-α production (Fig. 1(h)).

### PU.1 directly bound to and transactivated the *Syk* promoter

**Figure 2:**
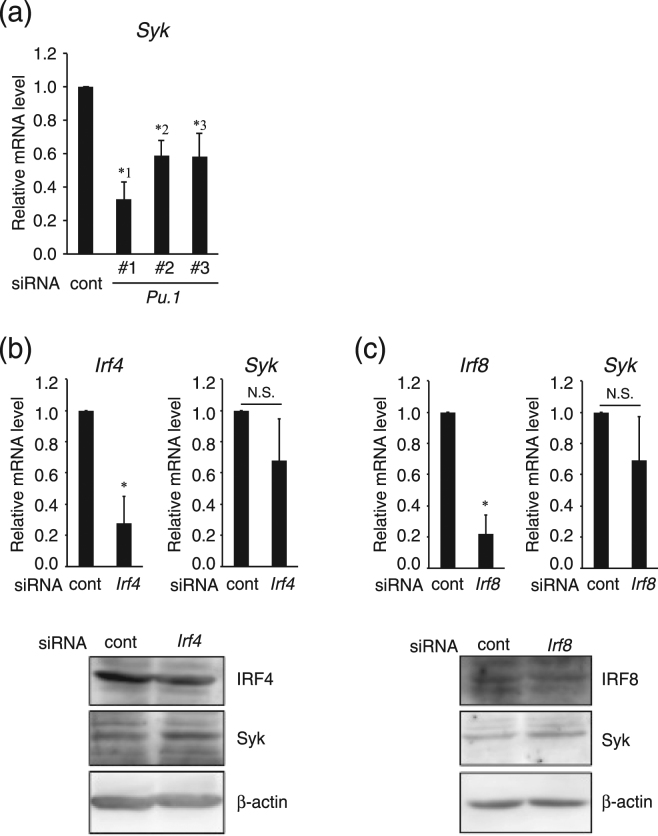
Effects of knockdown of PU.1, IRF4, and IRF8 on Syk expression. Relative mRNA levels of siRNA-target genes and Syk in siRNA transfectants. BMMCs were transfected with siRNA for PU.1 and its control (**a**), for IRF4 and its control (**b**), and for IRF8 and its control (**c**). Data are expressed as the ratio of the expression level of the respective control siRNA-introduced cells (cont in each graph). Western blotting profiles of IRF4 and IRF8 knockdown cells are shown at the bottom of Fig. 2(b) and (c), respectively. Full-length blots are included in Supplemental Fig. S2. The data represent the mean ± SD of “n” times repeated independent experiments performed with duplicate samples. (**a**). *1, *p* = 0.0077; *2, *p* = 0.017; *3, *p* = 0.0099. n = 3–4. (**b**). *p = 0.025. n = 3. (**c**). *p = 0.0082. n = 3.

The abovementioned results suggest that PU.1 regulates the Syk expression in MCs. Although Syk plays a key role in signal transduction just downstream of the cell surface receptor, the regulatory mechanism of cell type-specific expression of Syk is largely unknown. Thus, we analyzed the role of PU.1 in Syk expression as follows. First, we excluded the possibility of an off-target effect by the three siRNAs. As shown in Fig. 2(a), the level of Syk mRNA in transfectants was decreased in parallel with the degree of PU.1 mRNA reduction (see Fig. 1(a)). This result demonstrates that suppression of the Syk transcription was due to PU.1 knockdown and not an off-target effect.

**Figure 3:**
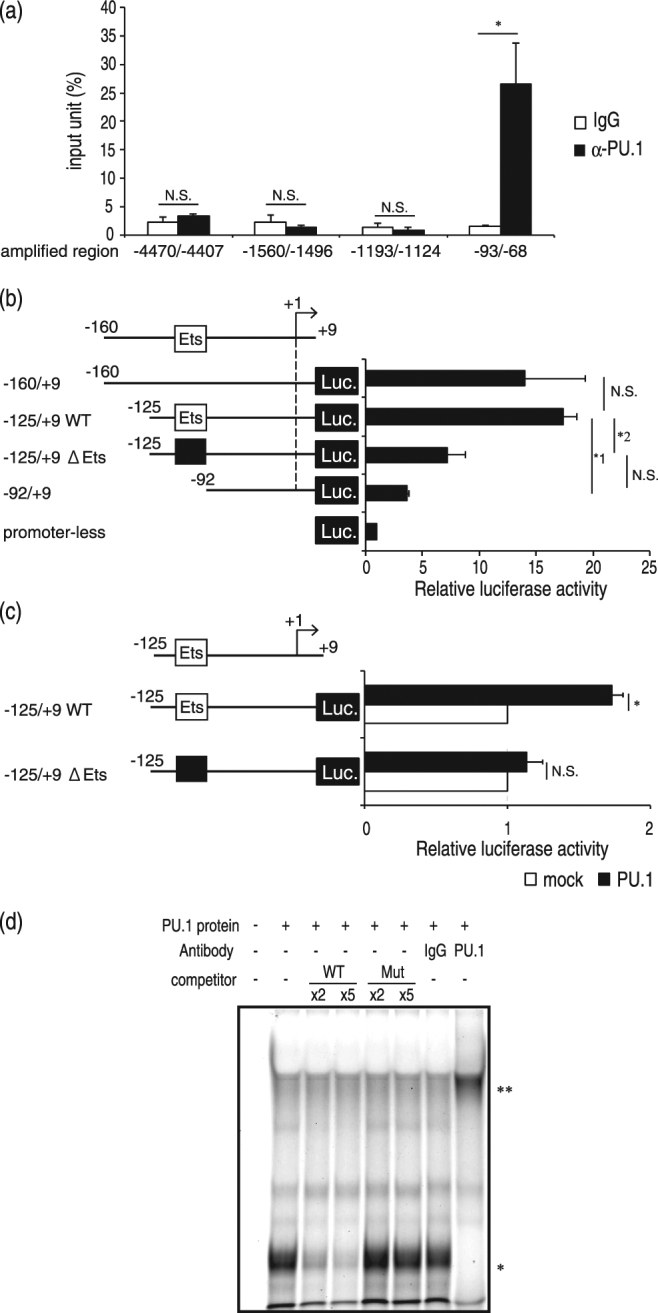
Involvement of PU.1 in the transactivation of the *Syk* promoter. The *Syk* promoter structure was analyzed by a ChIP assay (**a**), reporter assays (**b**) and (**c**), and EMSA (**d**). (**a**) ChIP assays were performed with control goat IgG (IgG) or anti-PU.1 Ab (α-PU.1). The amount of chromosomal DNA immunoprecipitated with control IgG (open bars) or anti-PU.1 Ab (closed bars) is shown. *p = 0.026; N.S., not significant. n = 3. (**b**) and (**c**) Relative luciferase activity is displayed as the ratio of luciferase activity versus that observed in cells transfected with promoter-less reporter plasmid **(b**) or mock vector (**c**). (**b**) *1, *p* = 0.0021; *2, *p* = 0.0013. n = 3. (**c**) *p = 0.012. n = 3. The data represent the mean ± SD of three independent experiments performed with duplicate samples (**a**), (**b**), and (**c**). (**d**) A typical result obtained from EMSA in one of three independent experiments. IgG, control goat IgG; PU.1, anti-PU.1 Ab. Competitive oligonucleotide with the wild-type sequence (WT) or the mutant sequence lacking Ets-motif (Mut) was added at two (x2)- or five (x5)-fold molar concentration of the probe DNA. Specific bands corresponding to the complex of the probe and PU.1 and a super-shift band corresponding to the complex of the probe, PU.1, and anti-PU.1 Ab are marked with an asterisk and double asterisks, respectively. A full-length gel with the lowest contrast is included in Supplemental Fig. S3.

PU.1 transactivates target genes in two ways: one is as a monomeric transcription factor, and the other is as a heterodimeric complex with the transcription factor IRF4 or IRF8. To investigate the role of IRF4 and IRF8 in Syk expression, we analyzed the effect of knockdown of IRF4 or IRF8 on Syk expression. Introduction of siRNAs for IRF4 and IRF8 significantly decreased the mRNA levels of IRF4 and IRF8, respectively. In this experimental condition, Syk mRNA and protein levels were maintained (Fig. 2(b) and (c)), indicating that IRF4 and IRF8 are not involved in Syk expression and that PU.1 activates the *Syk* promoter as a monomeric transcription factor. Then, we performed a chromatin immunoprecipitation (ChIP) assay to investigate whether PU.1 directly binds to the *Syk* promoter in MCs. As shown in Fig. 3(a), a significant amount of the chromosomal DNA containing the *Syk* minimum promoter was immunoprecipitated with anti-PU.1 Ab compared with the control Ab, whereas PU.1 binding was not detected further upstream. Next, luciferase assays using Syk promoter regions of various lengths were performed. As shown in Fig. 3b, the deletion between −125 and −93 markedly reduced luciferase activity in the MC line PT18. A nucleotide replacement at an Ets-motif (−100/−97) significantly decreased the luciferase activity of PT18 transfectants (Fig. 3(b)). Further luciferase assays with co-expression plasmids showed that exogenous expression of PU.1 transactivated the luciferase activity driven by the wild-type promoter but not that from the mutant promoter lacking the Ets-motif (Fig. 3(c)), suggesting that PU.1 transactivated the *Syk* promoter through the identified Ets-motif. Furthermore, we performed electrophoretic mobility shift assays (EMSAs) to confirm whether PU.1 directly binds to the Ets-motif at −100/−97 (Fig. 3(d)). A band shift appeared when the PU.1 protein was added to the reaction mixture containing probe DNA (lane 2). This band disappeared in the presence of excess amounts of a non-labeled wild-type competitor (lane 3, and 4), whereas the specific band remained when a mutant competitor lacking the Ets-motif was used instead of the wild-type competitor (lane 5, and 6), indicating that PU.1 bound to the *Syk* promoter via the Ets-motif. As the addition of anti-PU.1 Ab resulted in the disappearance of the specific band shift and the appearance of a new band showing lower mobility (lane 8), we confirmed that PU.1 was contained in this complex.

From these results, we concluded that PU.1 transactivates the *Syk* promoter by directly binding to the Ets-motif at −100/−97, which was identified as a critical *cis*-element.

### Involvement of PU.1 in the expression of Syk is commonly observed in dendritic cells (DCs) and human cells

**Figure 4:**
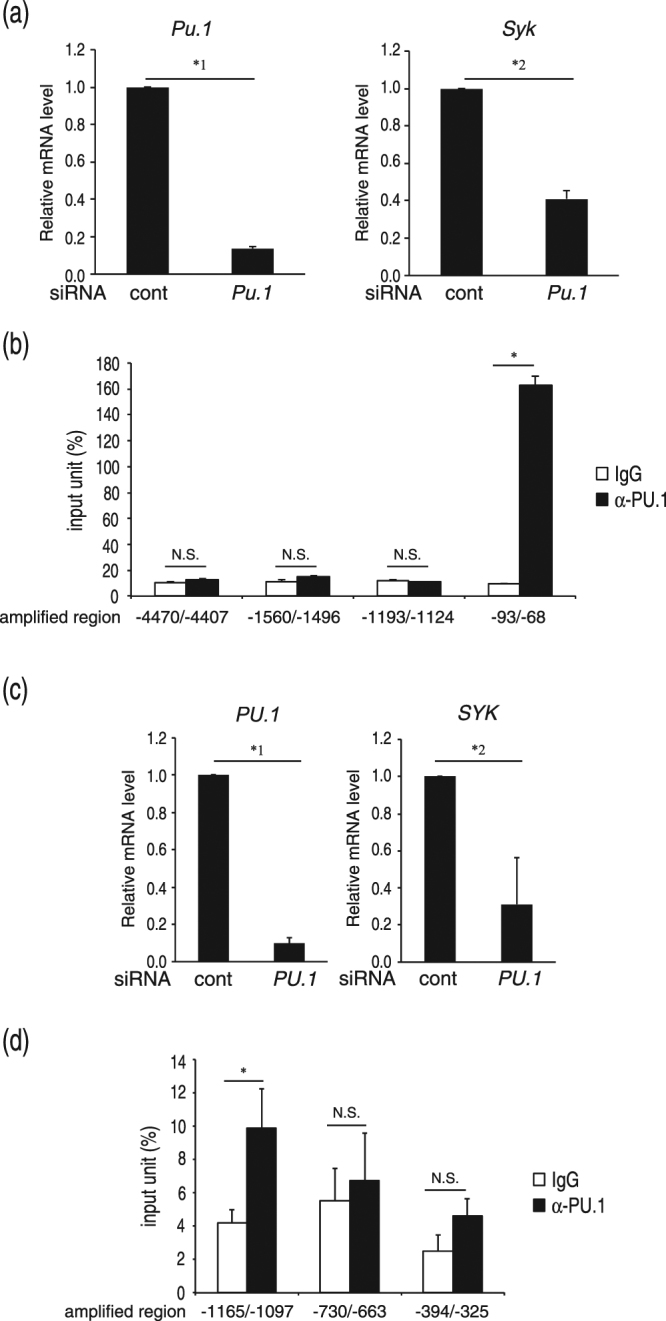
PU.1 is involved in the expression of Syk in mouse DCs and human MCs. (**a**) mRNA levels of PU.1 and Syk in siRNA-introduced BMDCs. *1, *p* = 0.0048; *2, *p* = 0.034. n = 3. (**b**) ChIP assay data demonstrating the amount of chromosomal DNA immunoprecipitated with anti-PU.1 Ab (α-PU.1) or control goat IgG (IgG) in BMDCs. *p = 0.0025; N.S., not significant. n = 3. (**c**) mRNA levels of PU.1 and Syk in siRNA-introduced LAD2 cells. *1, *p* = 0.00050; *2, *p* = 0.041. n = 3. (**d**) ChIP assay data demonstrating the amount of chromosomal DNA immunoprecipitated with anti-PU.1 Ab (α-PU.1) or control goat IgG (IgG) in LAD2 cells. *p = 0.041. n = 3. The data represent the mean ± SD of three independent experiments performed with duplicate samples.

Syk is expressed in other immune-related cells including B cells and monocytes, which play important roles in signal transduction from cell surface immunoreceptors, such as the B-cell receptor and C-type lectin family members. The apparent amount of PU.1 is detected in monocyte lineages and B cells. From these observations, we hypothesized that the *Syk* gene is transactivated by PU.1 not only in MCs but also in other lineages. To confirm this hypothesis, we determined the mRNA levels of PU.1 and Syk in PU.1 siRNA-treated BMDCs and found that the Syk mRNA levels were significantly reduced in PU.1 knockdown DCs (Fig. 4(a)). Furthermore, a ChIP assay showed that a significant amount of PU.1 binds to the above-identified region of the *Syk* promoter in DCs (Fig. 4(b)). Although IRF4 and IRF8 are also expressed in DCs, Syk expression was not affected by infection of IRF4 or IRF8 (data not shown), demonstrating that monomeric PU.1 transactivates the *Syk* gene in DCs in the same manner as that in MCs.

When we compared the nucleotide sequences of the mouse and human *Syk* genes, we found that the homology of the promoter sequences is not high and that the *cis*-enhancing element identified in the mouse gene was not observed in the human gene. Then, to investigate the role of PU.1 in human *SYK* gene expression, we transfected siRNAs for human PU.1 into the human MC line LAD2. As shown in Fig. 4(c), these siRNAs effectively knocked down human PU.1 mRNA levels and subsequently reduced Syk mRNA levels in LAD2 cells. In addition, a ChIP assay indicated that PU.1 bound to the proximal region of the human *SYK* gene in LAD2 (Fig. 4(d)).

From these results, we concluded that PU.1 is involved in Syk expression in MCs and DCs and in mice and humans.

### Effects of PU.1 knockdown or the immunomodulatory drug pomalidomide on *in vivo* function of MCs

**Figure 5:**
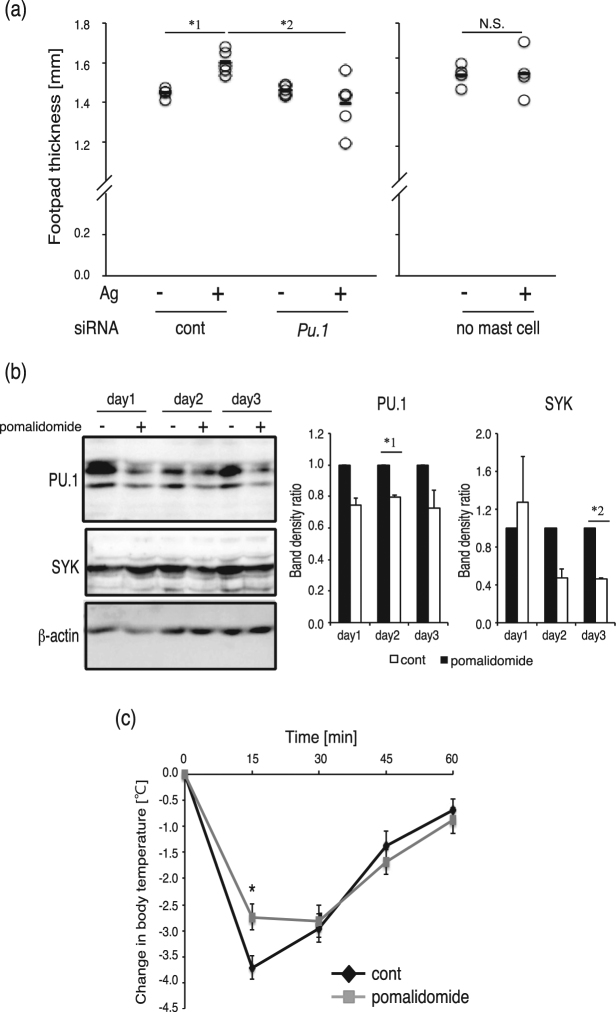
PU.1 siRNA and pomalidomide suppressed MC-mediated allergic responses *in vivo*. (**a**) The thickness of the mouse footpad before (−) and after (+) challenge with PBS containing TNP-BSA (Ag). PU.1 siRNA (PU.1)- and control siRNA (cont)-transfected BMMCs were pre-injected into the left and right footpads, respectively. *1, *p* = 0.00085; *2, *p* = 0.024. n = 5. (**b**) Western blotting analysis of whole cell lysates of LAD2 cells incubated in the presence (+) or absence (−) of pomalidomide for 1–3 days. A typical result is shown at left. Quantitative analysis data of the band density obtained in three independent experiments are shown at right. *1, *p* = 0.024; *2, *p* = 0.0079. Full-length blots are included in Supplemental Fig. S4. (**c**) IgE-mediated anaphylaxis in pomalidomide-administered mice. Rectal temperature was measured every 15 min. Data represent the mean ± SEM of individual mice (control; n = 12, pomalidomide; n = 11). *p = 0.0097.

To evaluate the effect of MC-specific knockdown of PU.1 on the *in vivo* response, we transfused BMMCs, in which PU.1 siRNA or its control was introduced, into the mouse footpad and determined the footpad thickness before and after an *i.v*. injection of antigen. The thickness of the control footpad after antigen injection was significantly greater than that before injection, whereas no thickening was observed in the footpad transfused with PU.1 knockdown cells (Fig. 5(a)). These results suggested that PU.1 knockdown is effective for suppression of MC-mediated responses *in vivo*.

Several studies have reported that the immunomodulatory drug pomalidomide down-regulates PU.1, which is one mechanism by which pomalidomide can be used to treat multiple myeloma^17–19^. To evaluate the effect of pomalidomide on expression and function of PU.1 in MCs, we treated LAD2 cells with pomalidomide. Western blotting analysis showed that the protein levels of PU.1 and Syk were decreased in LAD2 cells exposed to pomalidomide for 2–3 days (Fig. 5(b)). These results prompted us to investigate whether pomalidomide has a protective effect on the MC-mediated allergic response *in vivo*. Then, we analyzed the degree of passive systemic anaphylaxis, which is a well-established mouse model of the IgE-mediated reaction. As shown in Fig. 5(c), decreased body temperature due to the anaphylactic reaction was ameliorated by oral administration of pomalidomide.

These results suggest that down-regulation of PU.1 suppresses the MC-mediated *in vivo* allergic response.

## Discussion

Previously, we found that the transcription factor PU.1 transactivates the *FCER1A* gene (encoding FcεRIα) and that knockdown of PU.1 reduced expression of FcεRI and subsequently suppressed FcεRI-mediated activation of human MCs^15^. However, the role of PU.1 in the expression of signal transduction-related intercellular molecules in MCs was largely unknown. It was also unclear whether PU.1 knockdown suppresses FcεRI-mediated activation of mouse MCs, as observed in human MCs. To clarify these points, we evaluated the effect of PU.1 knockdown on the gene expression and function of mouse MCs *in vitro* and *in vivo*. PU.1 knockdown reduced cell surface expression of FcεRI and subsequently suppressed IgE-mediated activation of mouse MCs. Among various signal transduction-related molecules, Syk was identified as a target gene for PU.1. A series of promoter analyses showed that PU.1 directly transactivated the *Syk* gene. PU.1 knockdown using siRNA and an immunomodulatory drug suppressed MC activation *in vitro* and *in vivo*.

Syk is a non-receptor tyrosine kinase that plays an important role in signal transduction initiated by cell surface immunoreceptors, including BCR, FcεRI, FcγR, and C-type lectins^20^. In the present study, we showed that PU.1 is critical for expression of Syk as a transcactivator that directly targets the *Syk* gene in MCs and DCs. PU.1 expression is observed in hematopoietic cells, especially monocytes, B cells, and neutrophils and is also detected in T cells and MCs. The cell-type specificity of Syk-expressing cells, such as B cells, MCs, neutrophils, macrophages, DCs, osteoclasts, and immature T cells, is similar to that of PU.1 expression. Although we used MCs and DCs to demonstrate the involvement of PU.1 in Syk expression, the role of PU.1 in Syk expression may be common in other hematopoietic lineages, such as B cells. Syk inhibitors exhibit therapeutic effects on allergic diseases, autoimmune diseases, and B lymphocyte malignancies^20^. Therefore, PU.1 suppression may be useful for preventing these diseases by inhibiting Syk-mediated signaling accompanied by suppressing the expression of several cell type-specific genes.

In addition to Syk reduction, PU.1 knockdown suppressed the expression of cell surface FcεRI due to a decrease in Ms4a2 mRNA (encoding FcεRIβ), along with a substantial increase in SHIP-1 and SHIP-2. In classical promoter analyses, GATA1 and FOG-1 were identified as transcriptional regulators of the mouse *Ms4a2* gene^9,16^, whereas Oct-1 and MZF-1 were originally identified as regulators of the human *MS4A2* gene^10,13^. This difference is likely because the nucleotide sequences of the promoters are not conserved between humans and mice. Recently, we demonstrated that the mRNA level of the human *MS4A2* gene was decreased in GATA2 siRNA-introduced MCs but was not affected by PU.1 siRNA^15^. Therefore, the down-regulation of Ms4a2 mRNA by PU.1 knockdown is a mouse-specific observation. Further detailed analysis is required to clarify the mechanism underlying PU.1 involvement in the transcription of the *Ms4a2* gene, for instance, as a direct transactivator or an indirect regulator through transcription of another factor. PU.1 knockdown has not been found to up-regulates SHIP-1 expression in any cells thus far. Interestingly, several transcription factors were identified as suppressive regulators of *SHIP* gene expression; for example, Ikaros binds to the promoter of the *SHIP-1* gene in B cells, and a deficiency of Ikaros up-regulates SHIP-1 expression^21^, whereas Fli-1 suppresses SHIP-1 transcription in erythroleukemia^22^. Although the molecular mechanism underlying how these hematopoietic cell-specific transcription factors function as suppressors of the *SHIP-1* gene is largely unknown, detailed analysis regarding of the role of PU.1 in SHIP-1 expression may clarify this issue. Regardless, we concluded that PU.1 knockdown suppressed FcεRI-mediated signaling with a decrease in positive regulators (Syk and FcεRI) and an increase in suppressors (SHIPs).

In the present study, we used two models to demonstrate that PU.1 knockdown suppressed *in vivo* allergic responses. In the first experiment, to evaluate the effect of MC-specific knockdown of PU.1, we transfused siRNA-pre-injected MCs into mice. Although MC-deficient mice, such as Wsh/sh, would be a better recipient to exclude the involvement of endogenous MCs, we showed a significant effect of PU.1 siRNA on the MC-mediated response *in vivo*, even in this experimental condition. As a preliminary experiment, we examined the degree of anaphylactic reaction of Wsh/sh mice injected with PU.1 siRNA-introduced MCs or control cells and found that the degree of rapid body temperature decrease was reduced by MC-specific knockdown of PU.1 (data not shown). For the second approach, we administered the immunomodulatory drug pomalidomide to mice. Although pomalidomide reduced the protein levels of PU.1 and Syk in human MC cells, several issues remain to be clarified, such as the possibility that pomalidomide modulates the expression of other molecules involved in the activation of MCs. In addition, whether the protein levels of PU.1 and Syk in MCs were reduced by the pomalidomide treatment should be examined. Further detailed analysis regarding the effect of pomalidomide on allergic responses is required.

We demonstrated that PU.1 knockdown significantly reduced the MC-mediated allergic response *in vivo* and *in vitro*. The development of nuclear medicine, which can specifically and effectively deliver siRNA or antisense oligonucleotide to target cells, is required to further evaluate the efficacy of PU.1 knockdown for the treatment of immune-related diseases. We will investigate drug delivery systems for nuclear medicine to specific immune cells in future studies.

## Materials and Methods

### Mice and cells

BMMCs were generated from bone marrow cells of C57BL/6 mice (Japan SLC, Hamamatsu, Japan) by maintenance in RPMI 1640 (Sigma-Aldrich, St. Louis, MO) supplemented with 10% heat-inactivated fetal calf serum, 100 U/mL penicillin, 100 μg/mL streptomycin, 100 μM 2-mercaptoethanol, 10 μM minimum essential medium nonessential amino acid solution, and 5 ng/mL of murine IL-3 (PeproTech, London, United Kingdom) at 37 °C for more than 5 weeks. BMDCs were obtained by 10-day culture of BM cells in the medium containing 20 ng/ml of murine GM-CSF (PeproTech) instead of IL-3 in the above-described medium for BMMCs. All animal experiments were performed in accordance with the approved guidelines of the Institutional Review Board of Tokyo University of Science, Tokyo, Japan. The Animal Care and Use Committees of Tokyo University of Science specifically approved this study. The human mast cell leukemia cell line LAD2 (kindly provided by Dr. Arnold Kirshenbaum)^23^, mouse mast cell line PT18, simian kidney cell line CV-1, and human embryonic kidney cell line HEK293T were maintained as previously described^15,16^.

### Introduction of siRNA into cells

Small interfering RNAs for mouse PU.1 (Spi1-MSS247676), human PU.1 (Spi1-HSS186060), mouse IRF4 (MSS205501), and mouse IRF8 (MSS236848) and control siRNA (Stealth RNAi Negative Universal Control Lo GC, Med GC, and Hi GC (#12935–200, 300, and 400)) were purchased from Invitrogen (Carlsbad, CA). BMMCs of 2 × 10^6^ (or 2 × 10^5^) were transfected with 10 (or 1) μl of 20 μmol/L siRNA with a Neon 100 μl kit (or a Neon 10 μl kit) using a Neon transfection system (Invitrogen) set at Program #5. Introduction of siRNA into LAD2 cells and BMDCs was performed as previously described^15,24^. Briefly, a Neon transfection system was used for LAD2 cells^15^, and a Mouse Macrophage Nucleofector kit and Nucleofector II (Lonza, Basel, Switzerland) were used for BMDCs^25,26^.

### Quantification of mRNA by real-time PCR

Total RNA prepared from cells with an RNeasy kit (QIAGEN, Hilden, Germany) was reverse-transcribed using a Rever Tra Ace qPCR RT kit (TOYOBO, Osaka, Japan) to synthesize cDNA. The mRNA levels were quantified using a StepOne Real-Time PCR system (Applied Biosystems) with TaqMan Gene Expression Assays (Applied Biosystems) #Mm01270606_m1 for mouse *Pu.1*, #Mm00438867_m1 for mouse *Fcer1a*, #Mm00442780_m1 for mouse *Ms4a2*, #Mm00438869_m1 for mouse *Fcer1g*, #Hs02786711_m1 for human *PU.1*, #4352339E for rodent glyceraldehyde-3-phosphate dehydrogenase (*Gapdh*), and #4326317E for human *GAPDH* and a THUNDERBIRD probe qPCR Mix (TOYOBO).

For analysis of mouse *Syk*, *Inpp5d*, *Inppld*, *Lyn*, *Plcg1*, *Plcg2*, *Fyn*, *Stat5b*, and human *SYK*, the following primers were used with THUNDERBIRD SYBR qPCR Mix: mouse *Syk* (forward primer: 5′-CTACTACAAGGCCCAGACCC-3′, and reverse primer: 5′-TGATGCATTCGGGGGCGTAC-3′), *Inpp5d* (forward primer: 5′-CCACCTATCGATTTGAAAGACTG-3′, and reverse primer: 5′-GAGACGAATTGAGATGTGACTCC-3′) mouse *Inppld* (forward primer: 5′-TGCAGTCAATATGGAACATCAAG-3′, and reverse primer: 5′-CGAGTAGTCTTCTCATTCCCTGA-3′) mouse *Lyn* (forward primer: 5′-GCAGGGCAGTTTGGGGAAGTC-3′, and reverse primer: 5′-ACAGACATGGTGCCGGGCTTG-3′) mouse *Plcg1* (forward primer: 5′-ACAAGCTGTGGAAGTGCTCTCTTTA-3′, and reverse primer: 5′-GCCATCATAGAGGCCAGCAT-3′) mouse *Plcg2* (forward primer: 5′-CGGCACCCAGTTTGTCCTCA-3′, and reverse primer: 5′-AGAGCCACTTCACCGCATCC-3′) mouse *Fyn* (forward primer: 5′-CGTGACCTCCATCCCGAACT-3′, and reverse primer: 5′-AACTCAGGTCATCTTCCGTCCGT-3′) mouse *Stat5b* (forward primer: 5′-CAGGTGGTCCCCGAGTTTGCA-3′, and reverse primer: 5′-CAGATCGAAGTCCCCATCGGTA-3′), human *SYK* (forward primer: 5′-GGCAGGAGAATCGCTTGAAC-3′, and reverse primer: 5′-GGAGTGCAGTGGCATGATCTT-3′).

### IgE-mediated activation of BMMCs

The degranulation degree was determined as follows. BMMCs (5 × 10^5^) were sensitized with 200 ng of anti-TNP mouse IgE (clone IgE-3, BD Bioscience, San Jose, CA) in 1 ml of medium for 2 h at 37 °C and resuspended in 1 ml of Tyrode’s buffer containing 3 ng of TNP-BSA (LSL, Tokyo, Japan) after washing with Tyrode’s buffer. Beta-hexosaminidase activity in the supernatant at 30 min after TNP-BSA-stimulation was determined as previously described^15^.

### Measurement of cytokine concentration

The concentration of TNF-α in the culture media at 3 h after FcεRI cross-linking was determined using an ELISA kit (#MTA00B, R&D Systems, Minneapolis, MN).

### ChIP assay

ChIP assays were performed as previously described using a ChIP Assay Kit (Upstate, Lake Placid, NY) according to the manufacturer’s instruction with slight modifications^14,27^. Anti-PU.1 Ab (#D-19, Santa Cruz Biotechnology, Santa Cruz, CA) and goat IgG (#02-6202, Invitrogen) were used for immunoprecipitation. The amount of precipitated DNA was determined by quantitative PCR using an Applied Biosystems StepOne real-time PCR system. The nucleotide sequences of the primer sets for PCR were as follows: mouse *Syk* promoter −68/+93 (forward primer; 5′-AAGTTCTCCGGAGGAGGAAG-3′, and reverse primer; 5′-AGGCGGAGTGGCTCCTAC-3′), −1193/−1124 (forward primer; 5′-TTAAAGTGACACGGGAATTAGTTAGC-3′, and reverse primer; 5′-GCCACCAGAGCCTTACACAGA-3′), −1560/−1496 (forward primer; 5′-CCTCTAGCCTCCACATGCATTT-3′, and reverse primer; 5′-GCAATATGCACACGTATGTGGAT-3′), −4470/−4407 (forward primer; 5′-GGCTGGAGACCAAGTGTTCAA-3′, and reverse primer; 5′-CCTGAGGCGCCTATTGTAATTT-3′), and human *SYK* promoter −394/−325 (forward primer; 5′-CCAGGGAATATGCCATGCA-3′, and reverse primer 5′-CACCCAGCGGCCCTTT-3′), −730/−663 (forward primer; 5′-AGGTCTGGATGCCGTTTTGT-3′, and reverse primer; 5′-CAACCCATCCCCCTTTTCC-3′), and −1165/−1097 (forward primer; 5′-GAAGGCAAAAGCCAACCTGTAA-3′, and reverse primer; 5′-GTGACATACAGAAATTGGAGGTAAGG-3′).

### Luciferase assay

A series of reporter plasmids carrying mouse Syk promoter regions of various lengths just upstream of the luciferase cDNA was generated based on the pGL-4 Basic vector (Promega, Madison, WI). A PrimeSTAR Mutagenesis basal kit (TaKaRa Bio, Shiga, Japan) was used to generate mutant reporter plasmids. The expression plasmid pCR3-mPU.1 was generated by ligation of mouse PU.1 cDNA amplified by PCR using total RNA from BMDCs as a template and the following oligonucleotides designed for the outsides of the initiation codon and termination codon, as forward and reverse primers, respectively (5′-ATCCGCCTTGATCCCCACCGAA-3′ and 5′-CGGGCGACGGGTTAATGCTATG-3′) into pCR3.1 (Invitrogen). The plasmid pCR3-mPU.1 or mock vector (pCR3.1) was introduced into cells with an internal control plasmid, pRL-CMV (Promega). Transfection of BMMCs and PT18 was performed using a Neon system in the same way as for siRNA transfection, and calcium phosphate was used for transfection of CV-1 and HEK293T cells. Briefly, plasmid DNA in 0.25 M CaCl_2_ solution was mixed with an equal volume of 2XHBSS, and the mixture was added dropwise to the cells after incubation for 20 min at room temperature. Determination of luciferase activity was performed as previously described using a 1420 Luminescence Counter ARVO Light (Perkin Elmer)^25^. For determination of β-galactosidase activity, cell lysates were incubated with a substrate, *o*-nitrophenyl-β-D-galactopyranoside (ONPG) (Thermo Scientific, Waltham, MA), for the appropriate time at 37 °C, and the absorbance of the reaction mixture at 405 nm was measured.

### EMSA

EMSA was performed as previously described^28,29^. Fluorescence was detected using an image analyzer, Typhoon FLA7000 (GE Healthcare).

### Western blot analysis

Western blot analyses were performed as described previously^24,28^. Antibodies against Syk (#LR), FcεRIβ (#N-18), IRF4 (#M-17), and IRF8 (#C-19) were purchased from Santa Cruz Biotechnology, and anti-β-actin Ab (#AC-15) was from Sigma-Aldrich. Anti-PU.1 Ab was the same as that used in ChIP assays.

### Flow cytometry

The cell surface expression level of mouse FcεRI was determined by a MACS Quant (Miltenyi Biotech, Tubingen, Germany) using an anti-mouse FcεRIα Ab (MAR-1, eBioscience). For intracellular staining, a Foxp3/Transcription Factor Staining Buffer Kit (TONBO) was used.

### IgE-mediated i*n vivo* response

PU.1 siRNA- or negative control siRNA-transfected BMMCs were sensitized with 0.2 mg/ml IgE. After the cells were washed with PBS, they were injected into the left (PU.1 siRNA) and right (control siRNA) footpads of 8–9-week-old female mice. Five hours later, mice were injected intravenously with 200 μg of TNP-BSA. Footpad thickness was measured just before and 1 h after injection of TNP-BSA.

### Passive systemic anaphylaxis

Mice were orally administered 10 mg/kg/day of pomalidomide or saline for 6 days. On day 6, mice were injected intravenously with 3 μg/ml of TNP-specific IgE in 200 μl of saline and then injected with 200 μg of TNP-BSA intravenously at 5 h after IgE injection. The body temperature of each animal was measured every 15 min for 1 h after antigen injection.

### Statistical analysis

Statistical analysis was performed using a two-tailed Student’s t-test with *p* values < 0.05 considered to be significant.

## Electronic supplementary material

Supplemental Figures
