# Supplementary material for: The effect of PU.1 knockdown on gene expression and function of mast cells
Source: Sci Rep. 2018 Jan 31;8:2005. doi: 10.1038/s41598-018-19378-y (PMC5792452; doi:10.1038/s41598-018-19378-y)
Supplement: Supplementary file 1 — Supplemental Figures [file 41598_2018_19378_MOESM1_ESM.pdf]

The effect of PU.1 knockdown on gene expression and function of mast cells

Yoshihito Oda, Kazumi Kasakura, Izumi Fujigaki, Azusa Kageyama,

Ko Okumura, Hideoki Ogawa, Takuya Yashiro, and Chiharu Nishiyama

Department of Biological Science and Technology,

Faculty of Industrial Science and Technology, Tokyo University of Science,

6-3-1 Niijuku, Katsushika-ku, Tokyo 125-8585, Japan

Atopy (Allergy) Research Center, Juntendo University School of Medicine, 2-1-1

Hongo, Bunkyo-ku, Tokyo 113-8421, Japan

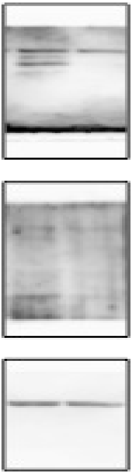

Full-length blots stained by anti-PU.1 Ab (top), anti-Syk Ab (middle), and anti-actin Ab (bottom), whose cropped profiles were shown in Fig. 1E.

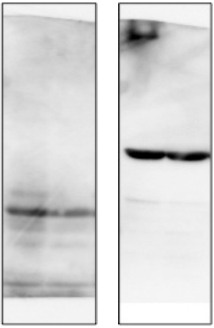

Full-length blots stained by anti-FcεRIβ Ab (left), anti-actin Ab (right), whose cropped profiles were shown in Fig. 1E.

Supplemental Fig. S1. Oda, Y.

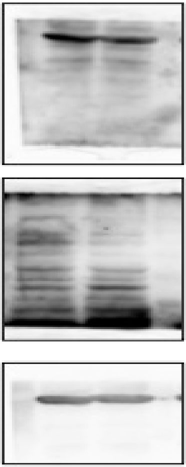

Full-length blots stained by anti-IRF4 Ab (top), anti-Syk Ab (middle), and anti-actin Ab (bottom), whose cropped profiles were shown in Fig. 2B.

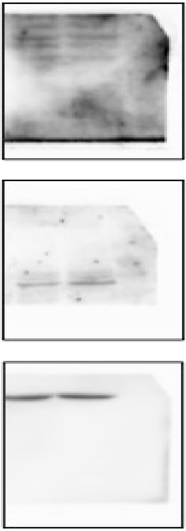

Full-length blots stained by anti-IRF8 Ab (top), anti-Syk Ab (middle), and anti-actin Ab (bottom), whose cropped profiles were shown in Fig. 2C.

Supplemental Fig. S2. Oda, Y.

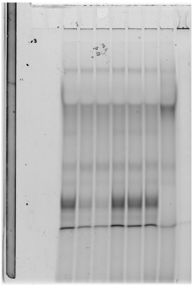

Full-length with the lowest-contrast gel profile of EMSA data (Fig. 3D).

Supplemental Fig. S3. Oda, Y.

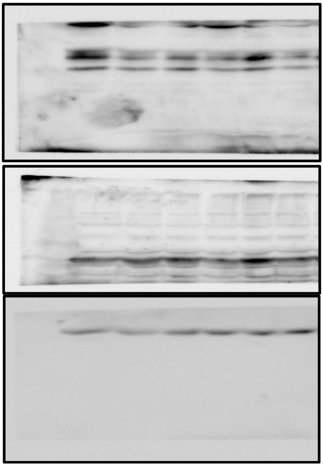

Full-length blots stained by anti-PU.1 Ab (top), anti-Syk Ab (middle), and anti-actin Ab (bottom), whose cropped profiles were shown in Fig. 5B.

Supplemental Fig. S4. Oda, Y.
